# A randomised controlled trial on hypolipidemic effects of *Nigella Sativa* seeds powder in menopausal women

**DOI:** 10.1186/1479-5876-12-82

**Published:** 2014-03-31

**Authors:** Ramlah Mohamad Ibrahim, Nurul Syima Hamdan, Rozi Mahmud, Mustapha Umar Imam, Suraini Mohd Saini, Saiful Nizam Abd Rashid, Siti Aisyah Abd Ghafar, Latiffah Ab Latiff, Maznah Ismail

**Affiliations:** 1 Department of Nutrition and Dietetics, Faculty of Medicine and Health Sciences, Universiti Putra Malaysia, 43400 Serdang, Selangor Darul Ehsan, Malaysia; 2 Department of Imaging, Faculty of Medicine and Health Sciences, Universiti Putra Malaysia, 43400 Serdang, Selangor Darul Ehsan, Malaysia; 3 Laboratory of Molecular Biomedicine, Institute of Bioscience, Universiti Putra Malaysia, 43400 Serdang, Selangor Darul Ehsan, Malaysia; 4 Department of Community Health, Faculty of Medicine and Health Sciences, Universiti Putra Malaysia, 43400 Serdang, Selangor Darul Ehsan, Malaysia

**Keywords:** Clinical trial, Complementary medicine, Hyperlipidemia, Menopause, Nigella sativa, Plant bioresources

## Abstract

**Background:**

The risk of cardiovascular diseases (CVD) is increased tremendously among menopausal women, and there is an increasing demand for alternative therapies for managing factors like dyslipidemia that contribute to CVD development.

**Methods:**

In this study, *Nigella sativa* was evaluated for its hypolipidemic effects among menopausal women. In a randomised trial, hyperlipidemic menopausal women were assigned to treatment (n = 19) or placebo groups (n = 18), and given *N. sativa* or placebo for two months after their informed consents were sought. At baseline, blood samples were taken and at one month intervals thereafter until one month after the end of the study.

**Results:**

The results showed that *N. sativa* significantly improved lipid profiles of menopausal women (decreased total cholesterol, low density lipoprotein cholesterol and triglyceride, and increased high density lipoprotein cholesterol) more than the placebo treatment over 2 months of intervention. One month after cessation of treatment, the lipid profiles in the *N. sativa*-treated group tended to change towards the pretreatment levels.

**Conclusions:**

*N. sativa* is thought to have multiple mechanisms of action and is cost-effective. Therefore, it could be used by menopausal women to remedy hypercholesterolemia, with likely more benefits than with single pharmacological agents that may cause side effects. The use of *N. sativa* as an alternative therapy for hypercholesterolemia could have profound impact on the management of CVD among menopausal women especially in countries where it is readily available.

## Background

The incidence of cardiovascular diseases (CVD) rises significantly in women when they attain menopause. Also, CVD continues to cause significant morbidity and mortality in those women [1,2]. In fact, the incidence of CVD is higher in menopausal women compared to women of other age groups and men of similar ages [3]. CVD is projected to remain the single leading cause of death globally and by 2020 almost 23.6 million people are projected to die from CVD particularly heart disease and stroke (WHO Death Estimated Report, 2011) [4] with significant impacts on the lives of women. The increase in CVD risk among menopausal women is mostly due to estrogen deprivation, which directly affects CVD risk factors such as dyslipidemia, diabetes mellitus, overweight, or hypertension at the time of menopause [5]. Dyslipidemia arises as a result of abnormal blood lipid profiles including especially those of high density lipoprotein (HDL), low density lipoprotein (LDL), and triglycerides (TG) [6,7], and remains one of the most important factors responsible for increased CVD risk among menopausal women. In Malaysia, CVD was reported to account for 25% of all deaths in 2008 (NCVD ACS Registry 4^th^ Annual Report, 2011) and dyslipidemia alone, or in combination with hypertention, or with diabetes were reported to be the main factors responsible [8]. Particularly, it is reported that an increasing number of Malaysian women are suffering from CVD, likely due to rising standards of living, increasing longevity and better health care, which ensures detection of more cases [9]. The use of pharmacological agents remains the standard treatment option for dyslipidemia, however, problems of side effects have necessitated the search for better options [10].

Although it is a physiological transition in a woman’s life, menopause is accompanied by problems, and hence women at this age must be managed appropriately for any health risks or problems, to ensure a fulfilling life. Nowadays, there is an increasing demand for plant bioresources in the management of chronic diseases like CVD, instead of synthetic drugs, to avoid side effects [11]. Moreover, traditional medicinal plants are often cheaper, locally available, and easily consumable (raw or as simple medicinal preparations). In line with this, *Nigella sativa* commonly known as black seed has been widely used in many Asian, Middle Eastern and Far Eastern countries as a spice and food preservative as well as a health remedy in traditional medicine for the treatment of numerous disorders. This plant has been a great focus of research and has several traditional uses and consequently has been studied extensively for its therapeutic properties [12]. Its medicinal benefits result from its rich and diverse chemical composition including amino acids, proteins, carbohydrates, and crude fiber, oils (fixed oil with 36-38% composed of unsaturated fatty acids), minerals, alkaloids, saponin and others [13]. Furthermore, many studies have shown good potential of hypoglycaemia, hypocholesterolemia and antioxidant effects of *N. Sativa*, which contribute to its cardioprotective effects [14-16]. However, little information exists concerning the effects of *N. sativa* on lipid parameters of menopausal women. Thus, we studied the effects of *N. sativa* seed powder on serum cholesterol, HDL-and LDL-cholesterol and TG in menopausal women for possible hypolipidemic activity.

## Materials and method

### Formulation of *N. sativa* and placebo capsules

Seeds of *N. sativa* were imported from Iran through a local company named Sari Tani Desa SDN BHD located in Shah Alam, Malaysia. Voucher specimens of seeds were kept at the Laboratory of Molecular Biomedicine, Institute of Bioscience, Universiti Putra Malaysia and the seed was identified and authenticated by Professor Dr. Maznah Ismail, Head of the Laboratory of Molecular Biomedicine, Institute of Bioscience, Universiti Putra Malaysia, Malaysia. Further, *N. sativa* seeds were processed into pharmaceutical grade capsules containing *N. sativa* powder and bottled according to Good Manufacturing Practices (GMP No.: MALLP20121468). Each capsule was prepared containing 500 mg powder of *N. Sativa*, and each bottle contained 60 capsules, stock for a month period to help with compliance of the respondents. While for the placebo, we used wheat germ which was bought from the same company, Sari Tani Desa Sdn. Bhd. and the entire process of wheat germ capsulation was same as *N. sativa* seeds, however with lesser dose of 100 mg per capsule.

### Chemicals

All chemicals used in this study were of analytical grade, and procured from Fisher Scientific (Loughborough, UK) and Merck (Darmstadt, Germany). Reagents and diagnostics kits used for TC, LDL, HDL and TG were supplied by Randox Laboratories Limited (Crumlin, County Antrim, UK).

### Study subjects and protocol

This clinical trial was conducted at Pusat Kesihatan Universiti (PKU), Universiti Putra Malaysia (UPM). Ethical clearance was obtained from the Faculty of Medicine and Health Sciences Medical Research Ethics Committee to conduct this study (approval number: JKEUPM/LECT_AUG [08]04), which aimed to evaluate the hypolipidemic effects of *N. sativa* in menopausal women. Thirty seven women (n = 37), who were menopausal for at least ≥ 12 months, within the age range of 45–60 years and who presented with moderate risk of hyperlipidemia were chosen as respondents in this study. Respondents were non-smokers, did not consume alcohol and did not have a history of any other chronic disease or drug/herb ingestion. Those with any of the features above were excluded from the study. These subjects were recruited among the university’s staff or ex-staff based on a health screening. Each qualified subject was informed about the study verbally and in writing according to Good Clinical Practice. These selected subjects were randomized into 2 groups (treatment and placebo) as follows: treatment group received capsulated *N. sativa* powder (n = 19), while the placebo group (n = 18) received the placebo capsules. The physical and pathological histories of these subjects were recorded, and their baseline anthropometric parameters measured (Table 1). All subjects were requested to maintain their regular lifestyles including their dietary intake and physical activity. Capsules of *N. sativa* powder were orally administered at a dose of 1 g after breakfast every day for a period of two months. A follow- up assessment was done after the subjects completed two months treatment. Fasting venous blood (5 ml) was drawn from the subjects at baseline and at intervals during (1 month of treatment) and after the treatment period (at the end of 2 months of treatment and one month after the end of study) for further analyses. Furthermore, at each follow up interval, blood pressure measurements were taken (Table 2).

**Table 1:** Biodata and baseline anthropometric measurements of respondents

**Characteristics**	**Nigella sativa (n = 19)**	**Placebo (n = 18)**
**Age (mean years**)	53.22 ± 2.16	53.71 ± 3.57
**Body weight (kg)**	63.07 ± 8.73	64.85 ± 10.69
**BMI (kg/m**^**2**^**)**	27.18 ± 4.34	27.75 ± 4.38
**Fat percentage (%)**	37.39 ± 8.86	39.48 ± 8.00
**Duration of menopause (%)**		
1-5 years	89.48	94.44
6-10 years	10.52	5.56
**Ethnicity (%)**		
Malay	89.48	100
Chinese	5.26	0
Indian	5.26	0
**Marital status (%)**		
Married	94.74	88.89
Single/divorcee/widowed	5.26	11.11
**Educational level (%)**		
Primary/Secondary	5.26	11.11
Diploma	31.58	44.45
Degree	63.16	33.33
Professional	0	11.11
**Employment status (%)**		
Working	73.68	66.67
Housewives/Retiree	26.32	33.33

Data presented as mean ± SD for age, body weight, BMI and fat percentage, BMI: body mass index.

**Table 2:** **Treatment effects of** ***Nigella sativa*** **and placebo on fasting blood glucose (FBG) and blood pressure levels**

**Parameters**	**Baseline**		**1**^**st**^**month**		**2**^**nd**^**month**		**Follow up**	
	***N. sativa***	**Placebo**	***N. sativa***	**Placebo**	***N. sativa***	**Placebo**	***N. sativa***	**Placebo**
FBG (mmol/L)	6.37 ± 0.61*	5.94 ± 0.44	5.69 ± 0.43*	5.81 ± 0.31	5.03 ± 0.51*	5.73 ± 0.75	5.48 ± 0.42*	5.70 ± 0.66
SBP (mmHg)	129.33 ± 15.44	138.40 ± 18.90	121.80 ± 17.40	131.27 ± 19.22	124.53 ± 13.31	140.71 ± 11.85	125.84 ± 15.48	144.71 ± 19.84
DBP (mmHg)	77.13 ± 9.16	83.93 ± 15.73	76.00 ± 12.90	79.53 ± 13.83	75.53 ± 9.56	89.00 ± 12.53	75.98 ± 10.64	93.00 ± 15.16

Values are mean ± SD. There was a downward trend in the means of the groups for both *N. sativa* and placebo over 2 months of intervention but no significant differences were observed.

*Significant between *N. S* vs. placebo group at *p* < 0.05. SBP: systolic blood pressure; DBP: diastolic blood pressure.

### Biochemical analysis

Whole blood was collected in plain tubes and further centrifuged at 2500 rpm for 15 min under 25°C. Serum was collected for the analyses of TC, TG, HDL and LDL levels using commercial diagnostic kits (Randox Laboratories Limited, Crumlin, County Antrim, UK) on *Selectra XL* chemical analyzer (Vita Scientific, Dieren, the Netherlands). Fasting blood glucose was determined using a glucometer (Roche Diagnostics, Indianapolis, IN, USA).

### Statistical analysis

All experimental values are presented as means ± standard deviation (SD). Statistical analysis was performed using SPSS windows program version 19 (SPSS Institute, Inc., Chicago, IL, USA). The One-way Analysis of Variance (ANOVA) with Bonferroni correction was used for analysis of data. Difference was considered to be significant if p < 0.05.

## Results

**Figure 1:**
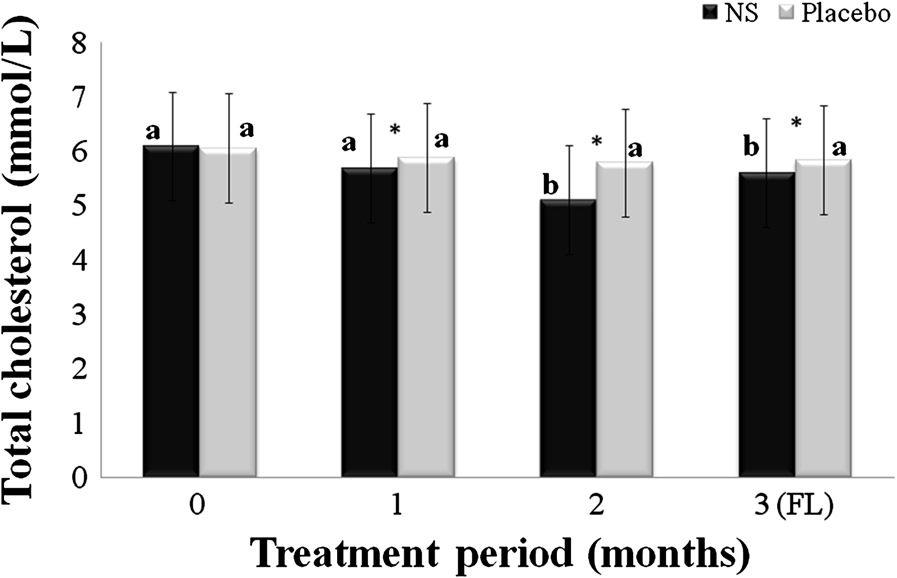
**Changes in serum total cholesterol (TC); menopausal women randomly assigned to *Nigella sativa *(N.S) or placebo groups were treated for 2 months and blood samples taken at baseline, and 1 and 2 months after the start of the intervention, and one month after cessation of intervention (FL), and used to evaluate serum TC.** Results are expressed as mean ± SD. *significant between *N. S* vs. placebo group at *p* < 0.05. Different letters on bars representing different intervals for the same group indicate significant difference between the intervals for the same treatment (*p* < 0.05).

**Figure 2:**
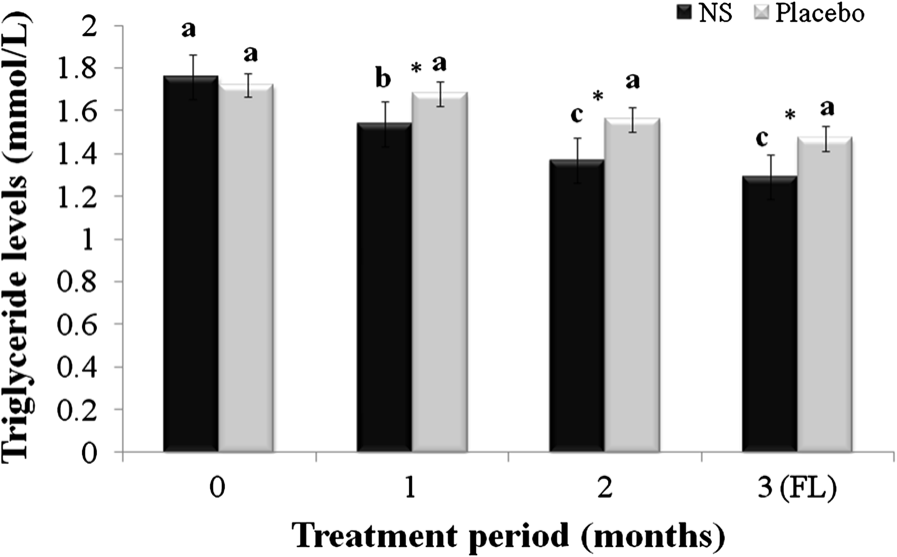
**Changes in serum triglyceride (TG); menopausal women randomly assigned to*Nigella sativa*(N.S) or placebo groups were treated for 2 months and blood samples taken at baseline, and 1 and 2 months after the start of the intervention, and one month after cessation of intervention (FL), and used to evaluate serum TG.** Results are expressed as mean ± SD. *significant between *N. S* vs. placebo group at *p* < 0.05. Different letters on bars representing different intervals for the same group indicate significant difference between the intervals for the same treatment (*p* < 0.05).

**Figure 3:**
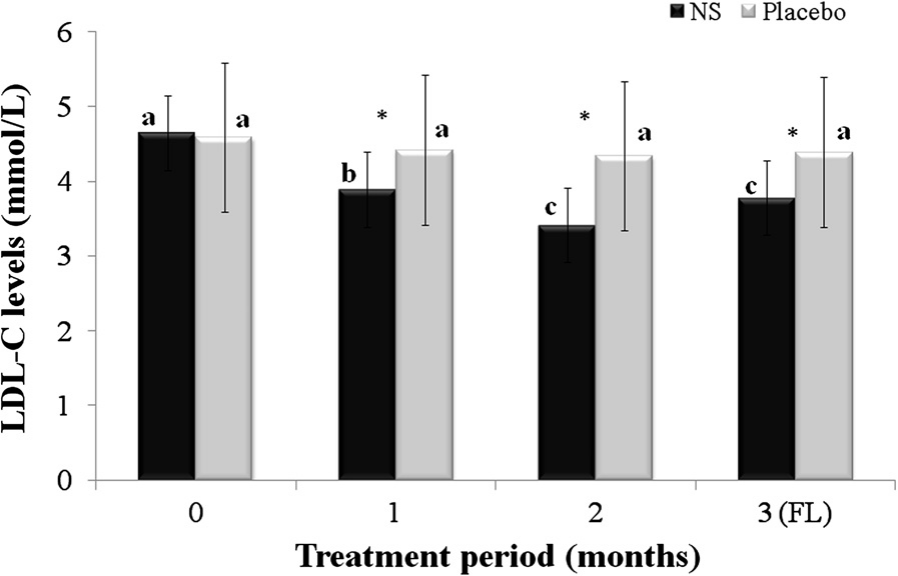
**Changes in serum low density lipoprotein (LDL); menopausal women randomly assigned to *Nigella sativa *(N.S) or placebo groups were treated for 2 months and blood samples taken at baseline, and 1 and 2 months after the start of the intervention, and one month after cessation of intervention (FL), and used to evaluate serum LDL.** Results are expressed as mean ± SD. *significant between *N. S* vs. placebo group at *p* < 0.05. Different letters on bars representing different intervals for the same group indicate significant difference between the intervals for the same treatment (*p* < 0.05).

**Figure 4:**
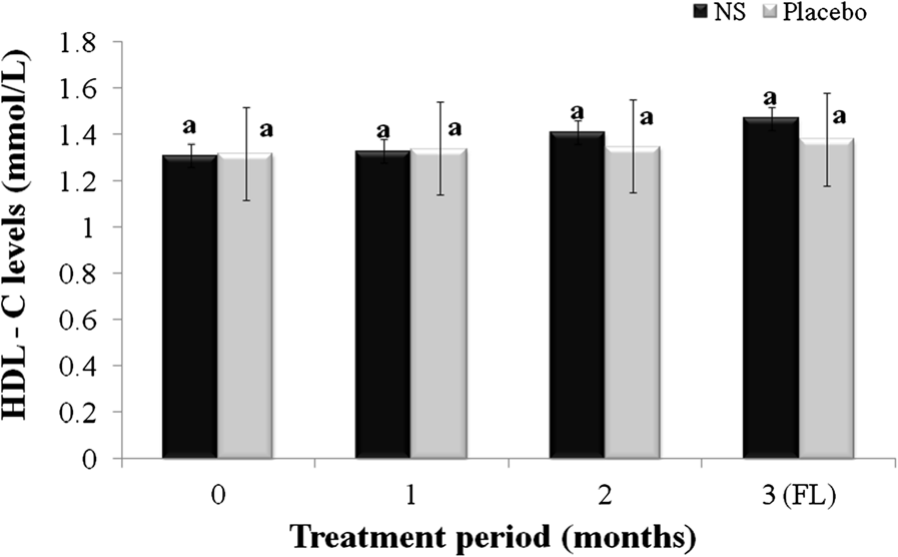
**Changes in serum high density lipoprotein (HDL); menopausal women randomly assigned to *Nigella sativa *(N.S) or placebo groups were treated for 2 months and blood samples taken at baseline, and 1 and 2 months after the start of the intervention, and one month after cessation of intervention (FL), and used to evaluate serum HDL.** Results are expressed as mean ± SD. *significant between *N. S* vs. placebo group at *p* < 0.05. Different letters on bars representing different intervals for the same group indicate significant difference between the intervals for the same treatment (*p* < 0.05).

This study was an attempt at determining the effects of *N. sativa* seeds powder on lipid profiles in humans especially among those with an increased risk of dyslipidemia (menopausal women). The baseline characteristic of the respondents in both treatment and placebo groups are presented in Table 1. Mean age of respondents in treatment and placebo groups participated in this study were 53.22 ± 2.16 and 53.71 ± 3.57, respectively. The changes in serum TC, TG, LDL and HDL are summarized in Figures 1, 2, 3, 4 for both treatment and placebo groups.

Mean TC of the *N. sativa* and placebo groups at baseline, 1^st^ and 2^nd^ months of treatment and one month after treatment ended (follow-up) were 6.09 ± 1.04, 5.69 ± 0.99, 5.11 ± 0.91, and 5.61 ± 0.79; placebo was 6.06 ± 1.02, 5.88 ± 0.77, 5.79 ± 0.68 and 5.84 ± 0.49 mmol/L, respectively. Mean TC of the respondents in the *N. sativa* group improved significantly, *p* < 0.05, by 16.09% at two months of treatment compared to baseline and significantly greater, *p* < 0.05, than that in placebo group.

Mean TG values of the *N. sativa* and placebo groups at baseline, 1^st^ and 2^nd^ months of treatment and follow-up were 1.76 ± 0.35, 1.54 ± 0.31, 1.37 ± 0.24, and 1.29 ± 0.35; 1.72 ± 0.53, 1.68 ± 0.46, 1.56 ± 0.24 and 1.47 ± 0.51 mmol/L, respectively. Similar trend of reduction in TG levels (22.16%) as TC levels was observed with two months treatment with *N. sativa* seeds powder which was significantly greater, *p* < 0.05, than the placebo group.

While, mean changes of LDL-C in the treatment groups at baseline, 1^st^ and 2^nd^ months of treatment and follow-up were 4.65 ± 0.89, 3.89 ± 0.83, 3.41 ± 0.76 and 3.78 ± 0.58; 4.59 ± 0.48 and 4.42 ± 0.55, 4.34 ± 0.61 and 4.39 ± 0.28 mmol/L, respectively. Reduction of LDL-C was 26.67% with significant changes, *p* < 0.05 with two months of treatment of *N. sativa* seeds powder in comparison to baseline. Again, the improvements in LDL-C throughout the treatment period were significantly greater, *p* < 0.05 in the *N. sativa* group compared to the placebo group.

Finally, HDL-C levels in both respective treatments at baseline, 1^st^ and 2^nd^ months of treatment and follow-up were 1.31 ± 0.22, 1.33 ± 0.30, 1.41 ± 0.25 and 1.47 ± 0.26; 1.32 ± 0.25, 1.34 ± 0.19, 1.35 ± 0.25 and 1.38 ± 0.27 mmol/L, respectively. HDL-C was found to have improved slightly in both groups, but no significant changes (*p* > 0.05) were observed between the two groups. Overall, improvements of lipid profile (TC, TG and LDL-C) throughout the treatment period were significantly greater, *p* < 0.05 in the *N. sativa* group compared to the placebo group except for HDL-C levels, which was similar to that produced by placebo.

Although the differences in blood pressures (Table 2) were not significant between the *N. sativa*-treated group and the control over the period of intervention, *N. sativa* produced better results and significant changes were observed in fasting blood glucose. The changes in these parameters also showed similar patterns to those of lipid profile; over the 2 months of intervention, the changes showed a downward trend but increased 1 month after cessation of intervention.

## Discussion

Menopause begins 12 months after a woman's final menstrual period and is associated with a dramatic drop in estrogen levels. This change in hormone levels often contributes to adverse metabolic changes that occur during the transitional pre- and post-menopausal periods, which make women prone to diseases. It has been proposed that estrogen hormone may be responsible for the protective effects seen amongst pre-menopausal women [17]. Estrogen exerts cardio-protective action by maintaining high levels of HDL and lower levels of LDL and TG. As can be recalled, loss of this protection after menopause may therefore be responsible for increased risk of developing CVD in menopausal women [18]. Moreover, cross-sectional studies have shown high TC, and LDL and TG levels are associated with menopausal status [19,20]. Longitudinal studies also showed increased TC, LDL, or TG levels were common at the time of menopause [21,22].

In this study, we determined the hypolipidemic effects of *N. sativa* in menopausal women as part of the increasing drive to provide better insights into the effects of plant bioresources on health, and their potential use for therapy of chronic diseases. Also, increasing numbers of women are thought to use complementary medicines for disorders associated with their menopause, and studies like these could provide evidence-based rationale for their choice of plant bioresources. Specifically, about 75% of women were reported to be using herbal and complementary medicines in their menopausal period in place of hormone replacement therapy (HRT) due to considerations on its side effects [23]. Furthermore, the baseline data from participants in the Study of Women’s Health Across the Nation (SWAN) indicated that 48.5% had used at least one complementary or alternative therapy during their menopausal years [24]. Moreover, *N. sativa* proved to have many beneficial health effects, particularly as hypolipidemic agent in various clinical trials [25,26]. To our knowledge, no clinical studies have examined the effect of *N. sativa* seed powder on hyperlipidemic menopausal women, while only a few have assessed the effect of *N. sativa* in overriectomized rats [27]. With the apparent lack of data on the effect of *N. sativa* in menopausal women, this study was necessitated to determine the protective effects of N. sativa on hyperlipidemic menopause women. The importance of this study is underlined by the fact that the overall cause of hyperlipidemia and the mechanisms leading up to its development in menopausal women differ considerable from in premenopausal women or men in general.

The results of the present study showed significant decrease in hyperlipidemia among menopausal women following treatment with *N. Sativa*, compared to the placebo group. The results also indicated a tendency for *N. sativa* to improve glycemia and blood pressure better than the placebo. Our results are in keeping with those of Inayat *et al*. (2009), who reported that oral treatment with *N. sativa* powder in hypercholesterolemic patients at the dose of 1 g daily for two months was found to reduce TC, LDL and TG, and increase HDL to highly significant extents [28]. Furthermore, TC, LDL and TG were reduced significantly after 6 months of treatment with *N. sativa* powder (500 mg) and statin in cardiac patients visiting Ch. Pervaiz Elahi Institute of Cardiology, Multan, Pakistan compared to placebo group receiving statin only [29]. In another study of 8 weeks at the Chamran Hospital (Isfahan, Iran), it was suggested that treatment with an oral *N. sativa* seed extract in male patients with mild hyperlipidemia and hypertension caused a significant dose-dependent decline in the levels of TC, TG and LDL relative to baseline data [30].

The hypolipidemic effects of *N. sativa* seeds powder does not seem to be due only to one component, but rather likely due to synergistic action of its different constituents including thymoquinone (TQ), migellamine, soluble fiber (mucilage), sterols, flavanoids and high content of polyunsaturated fatty acids (PUFAs) [31]. Additionally, in a previous study, we demonstrated that TQ in combination with other bioactive compounds in a formulation called TQ-rich fraction produced better regulation of cholesterol synthesis [32-34]. Multiple mechanisms of action may in fact contribute to the lipid-lowering effects of *N. sativa*. Earlier, we demonstrated that *N. sativa* was able to regulate cholesterol synthesis through regulation of HMG-CoA reductase, Apo-A1, Apo-B100 and LDL-receptor genes, an effect mediated by TQ and other *N. sativa* constituents [32-34]. In fact, our study had demonstrated that TQ in combination with other bioactive compounds weas better at regulation of cholesterol synthesis than TQ alone, suggesting that synergy of the compounds enhances the cholesterol-lowering effects of *N. sativa*[33,34]. In addition to the control of cholesterol synthesis we have demonstrated, other mechanisms of hypolipidemic action of TQ, the active components of *N. sativa*, have been proposed. TQ was shown to inhibit non-enzymatic lipid peroxidation in liposome and works as a scavenger of various reactive oxygen species including superoxide anion and hydroxyl radicals [32]. Antioxidants may also partly contribute to the overall functional effects of *N. Sativa*. Particularly, antioxidants (flavonoids) have been proposed to decrease cholesterol synthesis and suppress reactive oxygen species, nitrogen species formation and protect the antioxidant defence system [35]. Flavanoids are also thought to enhance the efficiency of liver cells to remove LDL from the blood circulation by increasing LDL receptor densities in the liver and binding to apolipoprotein B [36]. Lipid lowering effects of dietary soluble fibers [37] and sterols [38] in *N. sativa* could also contribute to decreased dietary cholesterol absorption, increased primary bile synthesis and its fecal losses. Besides, high content of PUFAs in *N. sativa* may also result in decreased serum TC [39,40].

## Conclusions

The present study showed that *N. sativa* caused significant decrease in TC, LDL and TG, and slight increase in HDL among menopausal women receiving *N. sativa* powder at a dose of 1 g daily for two months compared to placebo group. The study also demonstrated that *N. sativa* may improve other biochemical indices like glycemia and blood pressure in menopausal women. With an increasing demand for more alternative therapies for menopausal women due to concerns of side effects with pharmacological therapies, and considering that multiple factors play a role in the development and complication of hyperlipidemia among menopausal women, *N. sativa* may be used by hyperlipidemic menopausal women as a good complementary therapy. *N. sativa* is an easily available and cost-effective remedy to treat dyslipidemia, and its proposed multiple mechanisms of action may produce better hypolipidemic effects compared to standard pharmacological agents or other medicinal plants with a single mechanism of action. Studies like these contribute to better understanding of *N. sativa* effectiveness as a therapeutic agent, and could lead to its widespread use all over the world.

## Abbreviations

CVD: Cardiovascular diseases; HDL: High density lipoprotein; LDL: Low density lipoprotein; N.S: Nigella sativa; TC: Total cholesterol; TG: Triglyceride; TQ: Thymoquinone.

## Competing interests

The authors declared no conflict of interests.

## Authors’ contributions

RMI, NSH, MI and RM designed the study and together with MUI and SMS conducted the study, and biochemical and data analyses. RMI, MUI and SAAG drafted the manuscript, while RM, MI and LAL reviewed the manuscript and approved the final version for submission.
